# Long-term inorganic nitrate administration protects against ovariectomy-induced osteoporosis in rats

**DOI:** 10.17179/excli2022-5082

**Published:** 2022-08-24

**Authors:** Nasibeh Yousefzadeh, Sajad Jeddi, Khosrow Kashfi, Asghar Ghasemi

**Affiliations:** 1 Endocrine Physiology Research Center, Research Institute for Endocrine Sciences, Shahid Beheshti University of Medical Sciences, Tehran, Iran; 2 Department of Molecular, Cellular, and Biomedical Sciences, Sophie Davis School of Biomedical Education, City University of New York School of Medicine, NY, USA

**Keywords:** nitrate, nitric oxide deficiency, osteoporosis, oxidative stress, ovariectomy, rat

## Abstract

The risk of osteoporotic fractures increases in women after menopause. This study aims at determining the effects of long-term inorganic nitrate administration against ovariectomy-induced osteoporosis in rats. Rats were divided into 4 groups (n=6/group): Control, control+nitrate, ovariectomized (OVX), and OVX+nitrate. Sodium nitrate (100 mg/L in drinking water) was administered for 9 months. Trabecular bone quality in the proximal tibia was measured using a Micro-Computed Tomography (micro-CT) scanner at months 0, 1, 3, and 9. Levels of nitric oxide (NO) metabolites (NOx) and oxidative stress indices, and mRNA expression of endothelial NO synthase (eNOS) were measured at month 9 in the proximal tibia. Compared to controls, OVX rats had lower NOx levels by 47 %, eNOS mRNA expression by 55 %, catalase activity (CAT) by 45 %, total antioxidant capacity (TAC) by 70 %, and higher malondialdehyde (MDA) levels by 327 % in the bone tissue at month 9. OVX rats, compared to controls, had lower bone volume/tissue volume (BV/TV), trabecular number (Tb.N.), and trabecular thickness (Tb.Th.) by 32 %, 58 %, and 17 %, respectively, and higher trabecular separation (Tb.Sp.) by 123 %, at month 9. Nitrate administration to control rats increased TAC by 46 % in the bone tissue at month 9 but did not significantly affect other parameters in serum and bone tissue. Nitrate in OVX rats significantly increased NOx levels by 86 %, eNOS expression by 2.14-fold, CAT activity by 75 %, TAC by 170 %, and decreased MDA levels by 36 % at month 9 in the bone tissue. Nitrate-treated OVX rats at month 9 had higher BV/TV (42 %) and Tb.N. (61 %) and lower Tb.Sp. (15 %). Long-term inorganic nitrate administration at a low dose has protective effects against OVX-induced osteoporosis in rats; this effect is associated with increasing eNOS-derived NO and decreasing oxidative stress in the bone tissue.

## Introduction

Osteoporosis affects 30 % of women and 19 % of men worldwide and is a silent illness until experiencing a fracture (Migliorini et al., 2021[43]; Salari et al., 2021[63]). It has been estimated that osteoporotic fractures increase the risk of mortality (González‐Zabaleta et al., 2016[22]) and healthcare costs (by 200 %) until 2050 (Si et al., 2015[67]). In postmenopausal women, the risk of osteoporotic fractures increases by 1.5-3-fold (Vondracek et al., 2009[72]), and about 40 % of women will experience an osteoporotic fracture after menopause (ESHRE Capri Workshop Group, 2010[17]). Estrogen therapy decreases the risk of osteoporotic fractures in animals and humans; however, it also increases the risk of cancer and cardiovascular diseases (Rossouw et al., 2008[62]); therefore, new strategies are warranted.

Nitric oxide (NO) bioavailability decreases in bones of ovariectomized (OVX) rats (Mao et al., 2021[42]) and postmenopausal women (Jackson et al., 2006[28]) and is one of the primary mechanisms underlying osteoporosis (Jeddi et al., 2022[29]). The anti-osteoporotic effect of estradiol is partly mediated by NO derived from endothelial NO synthase (eNOS) (O'Shaughnessy et al., 2000[52]; Rajfer et al., 2019[59]), which is expressed in all bone cells (Hukkanen et al., 2003[27]). eNOS-derived NO decreases the risk of osteoporotic fracture by increasing osteoblast-mediated bone formation (Tai et al., 2007[70]) and decreasing osteoclast-mediated bone resorption (Wimalawansa, 2000[74]). eNOS^-/-^ rodents have lower osteoblast (Afzal et al., 2004[1]) and higher osteoclast (Kasten et al., 1994[31]) activities, as well as a lower ratio of osteoprotegerin (OPG)-to-receptor activator of NF-κB ligand (RANKL) (Percival et al., 1999[55]). These changes increase the risk of osteoporotic fractures in eNOS^-/-^ rodents by decreasing the quality of trabecular and cortical bone (Yan et al., 2010[81]). In addition, NOS inhibitors aggravate bone loss in OVX rats (Wimalawansa et al., 1996[73]; Wimalawansa, 2000[75]). These data imply that NO boosting may be a potential treatment for osteoporosis in postmenopausal women. 

The nitrate-nitrite-NO pathway is complementary to the NOS-independent pathway of NO production (Ghasemi, 2022[21]), and nitrate therapy is a strategy for NO boosting (Lundberg et al., 2015[39]). Inorganic nitrate, an NO donor, is converted to nitrite and then to NO; in addition to overcoming NO deficiency in postmenopausal women, NO has estradiol-like effects in the bone (Armour et al., 2001[4]) and, therefore, can decrease the risk of osteoporotic fractures. To our knowledge, only one study assessed the effects of inorganic nitrate on the bone microarchitecture in the tibia of OVX rats (Conley et al., 2017[15]); results indicate that short-term nitrate administration does not affect indices of trabecular bone quality in OVX rats. Different responses to short- and long-term administration of inorganic nitrate in animal studies have been reported; for example, inorganic nitrate decreases fasting glucose (Nyström et al., 2012[50]) and body weight (Bahadoran et al., 2020[7]) and improves diabetes-induced anemia (Khorasani et al., 2019[32]) only following long-term administration. Therefore, the current study's hypothesis is whether treatment with long-term (9 months) low-dose inorganic nitrate has favorable effects against ovariectomy-induced osteoporosis in Wistar rats.

## Materials and Methods

### Materials

Sodium nitrate, vanadium (III) chloride (VCl_3_), sulfanilamide, N-(1-Naphthyl) ethylenediamine dihydrochloride (NEDD), trichloroacetic acid (TCA), hydrochloric acid (HCl), sulfuric acid, zinc sulfate, sodium sulfate, hydrogen peroxide (H_2_O_2_), 5,5'-dithio-bis-(2-nitrobenzoic acid) (DTNB), ferrous sulfate, acetic acid glacial and dichromate potassium were purchased from Merck Company (Darmstadt, Germany). Thiobarbituric (TBA), tripyridyltriazine, 1, 1, 2, 3-tetra ethoxy propane, and reduced glutathione (GSH) were purchased from Sigma-Aldrich (Saint Louis, USA). N-butanol, iron chloride (FeCl_3), _and sodium pentobarbital were purchased from Fluka (Buchs, Switzerland), Biochem chemopharma (Cosne-Cours-sur-Loire, France), and Sigma-Aldrich (Hamburg, Germany) companies, respectively. In addition, TRIzol reagent and Master Mix 2X were obtained from Invitrogen (California, USA) and Thermo Fisher (California, USA) companies, respectively. Kits for measurement of estradiol and progesterone were obtained from Diagnostics Biochem company (Ontario, Canada), and for luteinizing hormone (LH), and follicle-stimulating hormone (FSH) were obtained from Cusabio Biotech (Wuhan, China) company.

### Animals 

In this study, female Wistar rats (n=24, 6-months-old) were obtained from the animal house of the Research Institute for Endocrine Sciences (RIES) of Shahid Beheshti University of Medical Sciences, Tehran, Iran. Rats were housed in polypropylene cages with a 12-h light (7 am to 7 pm) and 12-h dark (7 pm to 7 am) cycle at 23±2 °C with free access to tap water and regular food during the study. All procedures were performed according to the guideline for the care and use of laboratory animals in Iran (Ahmadi-Noorbakhsh et al., 2021[2]). In addition, the study protocol was approved by the Ethics committee of our institute (Ethic code: IR.SBMU.ENDOCRINE.REC.1398.111). 

### OVX-induced rat model of osteoporosis 

After overnight fasting, rats (200-220 g body weight) were anesthetized with sodium pentobarbital (60 mg/kg, IP), and ovaries were removed by the dorsolateral skin incision method (Yousefzadeh et al., 2020[83]). To confirm the ovariectomy, serum estradiol and progesterone levels were measured 2 months after ovariectomy using ELISA kits; the sensitivities of the assays were 10 pg/mL and 0.1 ng/mL, respectively. In addition, 2 months after ovariectomy, serum LH and FSH were also measured using rat-specific ELISA kits; sensitivities of the assays were 0.15 mIU/mL and 0.07 mIU/mL, respectively. Standard calibration curves of estradiol, progesterone, LH, and FSH are presented in Supplementary Figure 1. Intra-assay coefficients of variation (CVs) were <7 % in all assays. In addition, body weight and uterine weight were measured at month 9. To verify osteoporosis in the proximal tibia of OVX rats, bone volume/tissue volume (BV/TV, %), trabecular number (Tb.N., number/mm), trabecular thickness (Tb.Th., μm), and trabecular separation (Tb.Sp., μm) were measured 2 months after ovariectomy at month 0 using an *in vivo* X-ray Micro-Computed Tomography (micro-CT) scanner (LOTUS-in Vivo, Behin Ne-gareh Co., Tehran, Iran).

### Experimental design

**Figure 1:**
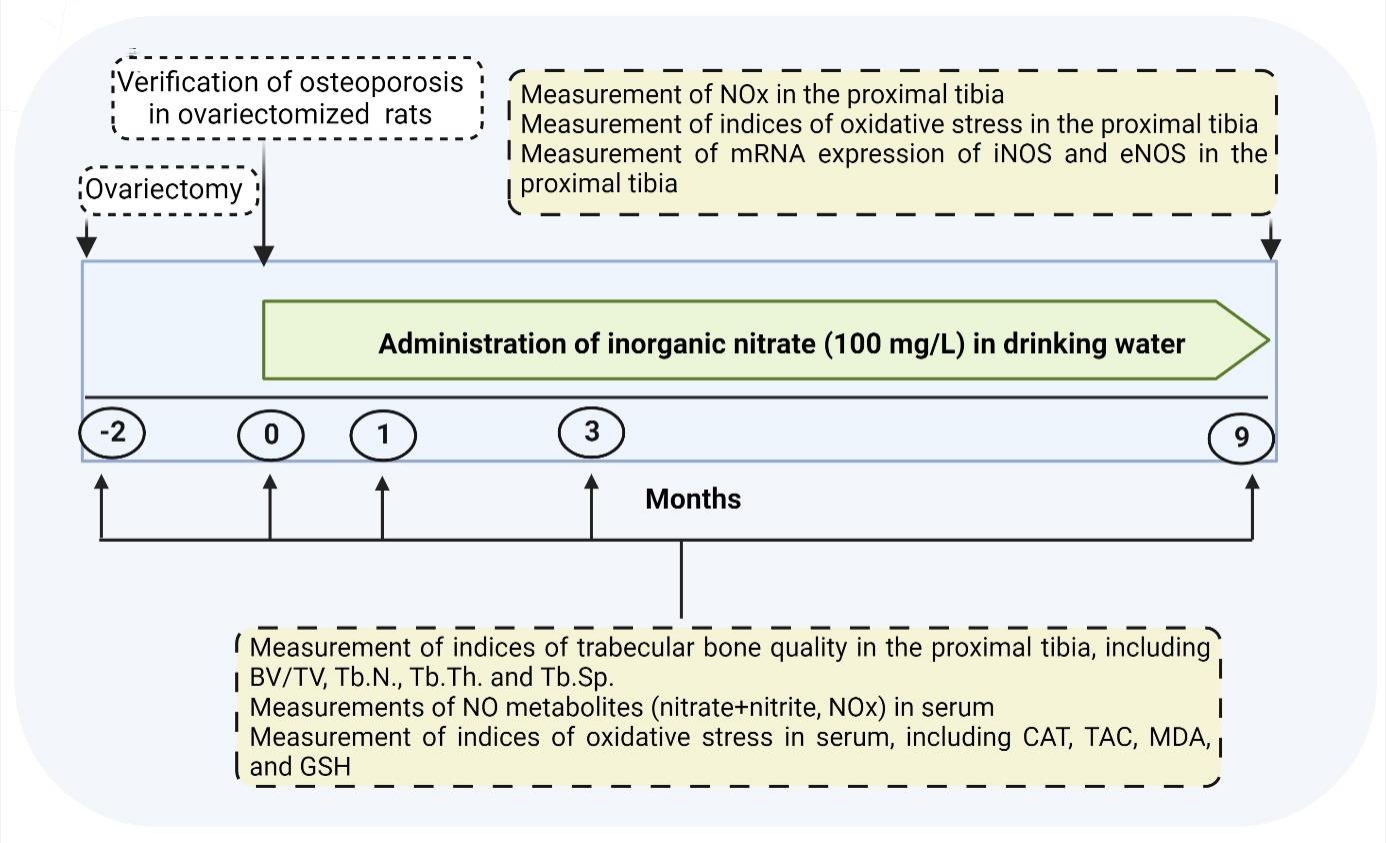
Experimental design of the study BV/TV, bone volume/tissue volume; CAT, catalase activity; eNOS, endothelial nitric oxide (NO) synthase; GSH, reduced glutathione; iNOS, inducible NOS; MDA, malondialdehyde; NOx, NO metabolites; TAC, total antioxidant capacity; Tb.N., trabecular number; Tb.Th., trabecular thickness; Tb.Sp., trabecular separation.

As shown in Figure 1(Fig. 1), after verifying osteoporosis in OVX rats at month 0, rats (8-months-old) were allocated to 4 groups (n=6/group): Control (without intervention), Control+nitrate (Control+N; without surgical intervention, and with nitrate administration at a dose of 100 mg/L for 9 months), OVX (with surgical intervention), and OVX+nitrate (OVX+N; with surgical intervention and nitrate administration at a dose of 100 mg/L for 9 months). We used a micro-CT scanner to measure indices of trabecular bone quality in the proximal tibia, including BV/TV, Tb.N., Tb.Th., and Tb.Sp. at months -2 (start of the study), 0 (start of nitrate administration), 1, 3, and 9 (the end of study and nitrate administration). Blood samples were collected from tail veins of anesthetized rats (inhalation of isoflurane) at months -2, 0, 1, 3, and 9, centrifuged (3000 g-10 min), and sera stored at -80 °C for measurements of NO metabolites (nitrate+nitrite, NOx), oxidative stress indices, including catalase activity (CAT), total antioxidant capacity (TAC), malondialdehyde (MDA), and GSH. In addition, at month 9, the left proximal tibia were removed from anesthetized rats (17-months-old) for measurement of NOx by the Griess method, oxidative stress indices, and gene expression of inducible NOS (iNOS) and eNOS, by real-time-PCR. As shown in Figure 1(Fig. 1), serum levels of NOx and indices for oxidative stress and trabecular bone quality were measured at months -2, 0, 1, 3, and 9. In addition, NOx and oxidative stress indices and expression of NOS enzymes in the bone tissue were measured at month 9.

### Micro-computed tomography

We used a micro-CT scanner to measure indices of trabecular bone (left proximal tibia) quality in anesthetized female rats at months -2, 0, 1, 3, and 9. The total scan duration was about 30 minutes for each anesthetized rat. The micro-CT scanner has a flat panel detector and a cone-beam micro-focus X-ray source. The X-ray tube voltage, current, and frame exposure time were set to 60 kV and 130 µA, and 1 second, respectively, to take high-quality images. LOTUS-in Vivo-ACQ software controlled all the protocol settings. BV/TV, Tb.N., Tb.Th., and Tb.Sp. indicate the volume of tissue occupied by trabecular bone, the number of trabeculae per unit of length, the average thickness of the individual trabeculae, and the average space between trabeculae, respectively (Supplementary Figure 2).

### Measurement of serum and bone tissue NOx and oxidative stress indices 

Blood samples were collected from rats' tail veins at months -2, 0, 1, 3, and 9 to measure serum NOx and oxidative stress indices. In addition, bone tissue samples were removed from anesthetized rats (17-months-old), frozen and dipped in liquid nitrogen, and pulverized using a porcelain mortar and pestle. A part of the bone powder (50 mg) was homogenized in 250 µL of phosphate-buffered saline (100 mM, pH 7.4, 1:5, w/v), centrifuged for 10 min at 10000 g at 4 °C; the supernatants of the homogenized bone tissues were then used for measuring NOx concentrations and oxidative stress indices.

NOx levels in serum and bone tissues were measured by the Griess method (Miranda et al., 2001[44]) with slight modification. In brief, supernatants and sera were deproteinized by zinc sulfate (15 mg/mL); 100 µL of deproteinized samples were then added to a microplate well; 100 µL of VCl_3_ (8 mg/mL in 1 M HCl), 50 µL of sulfanilamide (2 % in 5 % HCl), and 50 µL NEDD (0.1 % in ddH_2_O) were then added to each well. After incubation for 30 min at 37 °C, absorbance was read at 540 nm. Intra- and inter-assay CV were 1.8 % and 4.7 %, respectively. Sodium nitrate (range: 0-100 µM) was used to generate a standard calibration curve for determining NOx levels. NOx levels in serum and bone tissue are expressed as µM.

CAT activity in the serum and supernatants of the homogenized bone tissue were measured by the Hadwan method (Hadwan, 2016[24]). In brief, 20 µL of sera or supernatants of the homogenized bone tissues were added to test tubes, containing 200 µL of H_2_O_2 _reagent [335 µL H_2_O_2 _30 % in 50 mL sodium potassium phosphate buffer (50 mM, pH=7.4)] and control tubes, containing 200 µL of distilled water. In addition, the standard tube in the current test consisted of 20 µL of distilled water and 200 µL of H_2_O_2 _reagent. After incubation at 37 °C for 3 min, 400 µL of dichromate acetic acid reagent (60 mL acetic acid glacial added to 20 mL dichromate potassium 5 %) was dispensed into all tubes and incubated at 99 °C for 10 min. After cooling and centrifugation, the supernatant (300 µL) was read at a wavelength of 570 nm. In this method, dichromate acetic acid is reduced to chromic acetate in the presence of H_2_O_2 _at 99 °C; H_2_O_2 _concentration is directly proportional to the concentration of chromic acetate. CAT activity was calculated using the following formula: CAT activity (U/L) = 2.303/ (time of incubation)×Log [(absorbance of the standard tube)/(absorbance of the test tube - absorbance of the control tube)]×(total volume of reagents in the test tube)/(volume of the serum). Intra- and inter-assay CVs for CAT activity were 3.2 % and 3.6 %. CAT activity in serum and bone tissue is expressed as U/L.

TAC in serum and supernatants of the homogenized bone tissue was measured by the ferric reducing antioxidant power (FRAP) assay (Benzie and Strain, 1999[10]). In brief, 50 μL of samples were added to tubes with FRAP reagent (1500 μL, freshly prepared and pre-warmed at 37 °C) and incubated at 37 °C for 10 min. FRAP reagent contained 100 mL of acetate buffer (300 mM, pH=3.6), 10 mL of tripyridyltriazine (10 mM in 40 mM HCl), and 10 mL of FeCl_3 _(20 mM in distilled water). After 10 min, the absorbance was read at 593 nm. In this method, at low pH, an iron-containing tripyridyltriazine complex [(Fe3 +-TPTZ)] is reduced to the ferrous form, which has an intense blue color. Intra- and inter-assay CVs for TAC were 1.0 %, and 1.2 %. The present study used ferrous sulfate solutions (range: 0-200 µM) to generate standard calibration curves for TAC levels. TAC levels in serum and bone tissue are expressed as µM.

MDA levels in serum and supernatants of the homogenized bone tissue were measured by the Satoh method (Satoh, 1978[64]) with slight modifications. In brief, 100 µL of samples were added to the tubes containing 500 µL of TCA [20 % in distilled water (v/v)], and tubes were left to stand 10 min at room temperature. After centrifugation for 10 min, the supernatant was discarded, and the precipitate washed with sulfuric acid (0.05 M). Then 500 µL of sulfuric acid and 600 µL of TBA [0.67 % in sodium sulfate 2M (w/v)] added to this precipitate and incubated at 99 °C for 30 min. After cooling in cold water, 800 µL of n-butanol was added to each tube, vortexed, and centrifuged for 10 min, the absorbance of the pink-colored complex was read at 530 nm. Intra- and inter-assay CVs for MDA were 3.8 % and 4.4 %, respectively. In the present study, 1, 2, 3-tetra ethoxy propane (range: 0-20 μM) was used to construct standard calibration curves for determining MDA. MDA levels in serum and bone tissue are expressed as µM.

GSH level in serum and supernatants of the homogenized bone tissue was measured by the Sedlak and Lindsay method (Sedlak and Lindsay, 1968[65]) with slight modification (Rahman et al., 2006[58]). In brief, samples (20 μL) were added to tubes containing 200 μL of DTNB and incubated at 37 °C, and after 5 min, the absorbance was read at 412 nm. The assay is based on the reaction of GSH with DTNB (also known as Ellman's reagent) that produces the TNB chromophore, which has a maximal absorbance at 412 nm. The rate of formation of TNB, measured at 412 nm, is proportional to the concentration of GSH in the samples. Intra- and inter-assay CVs for GSH were 1.7 %, and 2.6 %, respectively. The present study used GSH (range: 0-100 µM) to construct standard calibration curve for determining GSH levels. GSH levels in serum and bone tissue are expressed as µM. Standard calibration curves of NOx, TAC, MDA, and GSH are presented in Supplementary Figure 3.

### Measurement of mRNA levels of target genes in bone tissues

At month 9, the left proximal bone was removed from anesthetized female rats and frozen at −80 °C until the mRNA expression of iNOS and eNOS was measured (Primer sequences are shown in Table 1(Tab. 1)). The frozen proximal tibia was dipped in liquid nitrogen and pulverized using a porcelain mortar and pestle. Total RNA was extracted from the powdered proximal tibia samples (10 mg) using TRIzol reagent. cDNA synthesis from extracted RNA and its amplification were done with SYBR Green PCR Master Mix 2X using a Rotor-Gene 6000 real-time PCR machine (Corbett, Life science, Sydney, Australia). The thermal cycling conditions included an initial denaturation (95 °C for 10 min) followed by 40 cycles (94 °C for 45 seconds, 58 °C for 45 seconds, and 72 °C for 1 minute) and a final extension (72 °C for 5 min). All samples were run in duplicate, and in the negative control reactions, nuclease-free water was used instead of templates.

### Statistical analyses

Data were analyzed using the GraphPad Prism software (Version 8). Two-way mixed (between-within) analysis of variance was used to compare serum NOx and oxidative stress levels and indices of trabecular bone quality in the proximal tibia at different times between groups. One-way analysis of variance was used to compare NOx levels and oxidative stress indices in the bone tissue. Bonferroni post-hoc test was used to determine the significance level among groups. Student t-test was used to compare serum levels of estradiol, progesterone, LH, FSH, uterine, and body weight in the control and OVX groups. Gene expressions relative to a reference sample were calculated based on cycle thresholds of target genes versus a reference gene (i.e., GAPDH) using REST software (Pfaffl et al., 2002[56]). Values are expressed as mean±SEM, and P-values < 0.05 were considered statistically significant.

## Results

### Verification of ovariectomy

Two months after ovariectomy at month 0, serum estradiol and progesterone levels were lower (P=0.037 and P=0.020, respectively), and serum levels of LH and FSH were higher (P=0.015 and P=0.024, respectively) than before ovariectomy (Table 2(Tab. 2)). In addition, OVX rats at month 9 had significantly lower uterine weight by 78 % and higher body weight by 21 % compared to controls.

### Verification of osteoporosis

As shown in Table 3(Tab. 3), at month 0, OVX rats had lower BV/TV by 19 % (P=0.096), Tb.N. by 33 % (P=0.004), Tb.Th. by 6 % (P=0.065) and higher Tb.Sp. by 93 % (P<0.001), indicating established osteoporosis in the proximal tibia of OVX rats.

### Verification of NO deficiency and efficacy of nitrate treatment in serum and bone tissue of OVX rats

**Figure 2:**
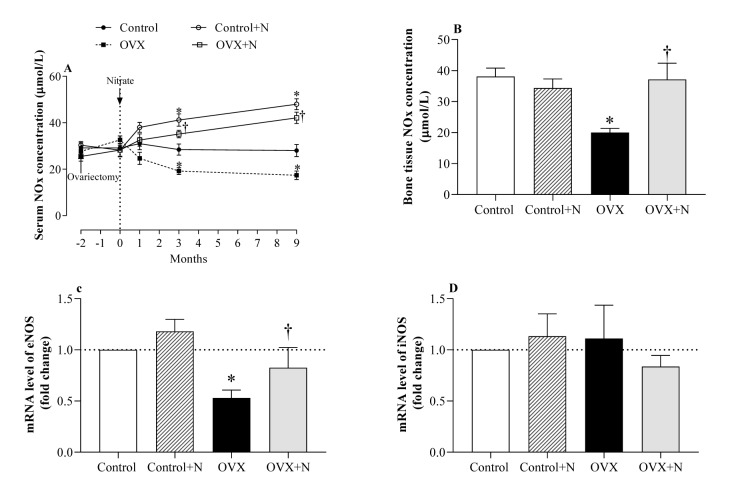
Effect of inorganic nitrate on (A) serum nitric oxide (NO) metabolites (NOx) during the study, (B) bone NOx levels, (C) bone mRNA expression of the endothelial NO synthesis (eNOS), and (D) inducible NOS (iNOS) at month 9 in ovariectomized (OVX) and control rats. ^*,† ^P < 0.05 compared to control and OVX rats, respectively. Results are mean ± SEM (n=6/group).

As shown in Figure 2A(Fig. 2), OVX rats had 32 % lower serum NOx levels at month 3 (P= 0.066) and 38 % lower at month 9 (P=0.049) compared to controls. In addition, at month 9, OVX rats had lower NOx levels in the bone tissue (20.0±1.6 vs. 38.1±2.7 µM, P=0.006, Figure 2B(Fig. 2)) and lower eNOS mRNA expression (55 %, P=0.005, Figure 2C(Fig. 2)) and comparable iNOS mRNA expression (Figure 2D(Fig. 2)) in the bone tissue than controls; these results indicate a deficiency of NO in the OVX rats.

Nitrate administration increased serum NOx concentrations in the control and OVX rats by 46 % (P = 0.034) and 83 % (P < 0.001) at month 3 and by 71 % (P = 0.001) and 143 % (P<0.001) at month 9, respectively (Figure 2A(Fig. 2)). In addition, nitrate administration to OVX rats increased bone NOx levels (86 %, P = 0.010) (Figure 2B(Fig. 2)) and bone eNOS expression (2.14-fold, P=0.085) (Figure 2C(Fig. 2)) but did not affect bone iNOS expression (Figure 2D(Fig. 2)). As shown in Figure 2(Fig. 2), nitrate administration to the control rats had no significant effects on bone NOx levels (Figure 2B(Fig. 2)), bone eNOS (Figure 2C(Fig. 2)), and bone iNOS expression (Figure 2D(Fig. 2)) at month 9.

### Effects of inorganic nitrate on indices of bone quality in the proximal tibia

**Figure 3:**
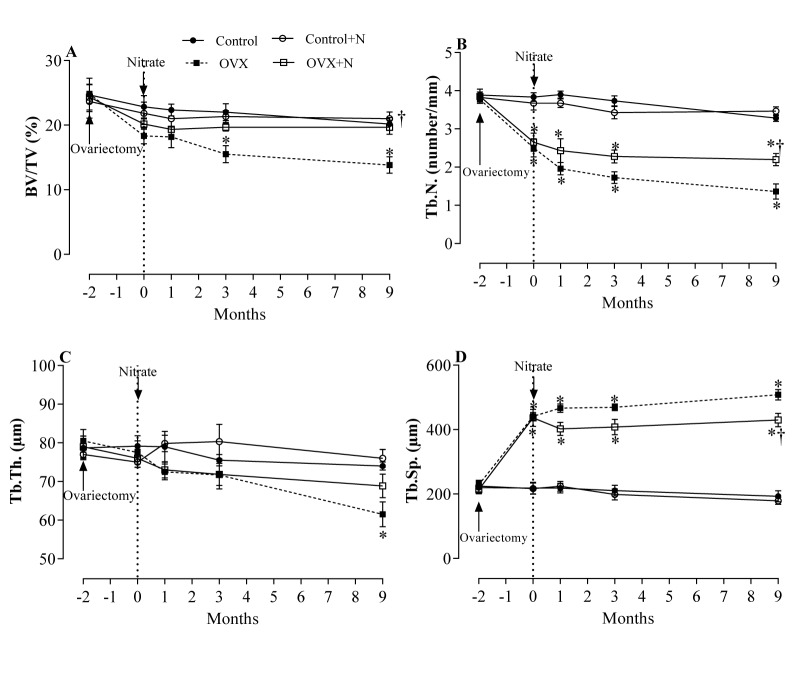
Effects of inorganic nitrate on trabecular bone volume (BV/TV, A), number (Tb.N., B), thickness (Tb.Th., C), and separation (Tb.Sp., D) in ovariectomized (OVX) and control rats. ^*, ^† P < 0.05 compared to control and OVX rats, respectively. Results are mean ± SEM (n=6/group).

OVX rats, compared to controls, had 50 % (P < 0.001) lower Tb.N. (Figure 3B(Fig. 3)) and 102 % (P < 0.001) higher Tb. Sp. (Figure 3D(Fig. 3)) 1 month after ovariectomy. At months 6 and 9, these changes were 54 % and 58 % lower for Tb.N. and 114 % and 123 % higher for Tb. Sp., respectively. In addition, compared to the controls, lower BV/TV at month 3 and month 9 (13.8 ± 1.3 vs. 20.2±0.9 %, P = 0.019) (Figure 3A(Fig. 3)) and lower Tb.Th. at month 9 (61.5 ± 3.2 vs. 74.0 ± 1.1 µm, P = 0.059) (Figure 3C(Fig. 3)) were observed in OVX rats. Nitrate-treated OVX compared to non-treated OVX rats, had 42 % (P = 0.037) higher BV/TV (Figure 3A(Fig. 3)) and 61 % (P = 0.002) higher Tb.N. (Figure 3B(Fig. 3)) respectively. Tb.Sp. was 15 % (P = 0.091) lower (Figure 3D(Fig. 3)), while Tb.Th. values were comparable at month 9 (Figure 3C(Fig. 3)). As shown in Figure 3A-D(Fig. 3), nitrate administration had no significant effect on BV/TV, Tb.N., Tb.Th., and Tb.Sp. in nitrate-treated control rats during the study period.

### Effect of inorganic nitrate on oxidative stress indices in serum

**Figure 4:**
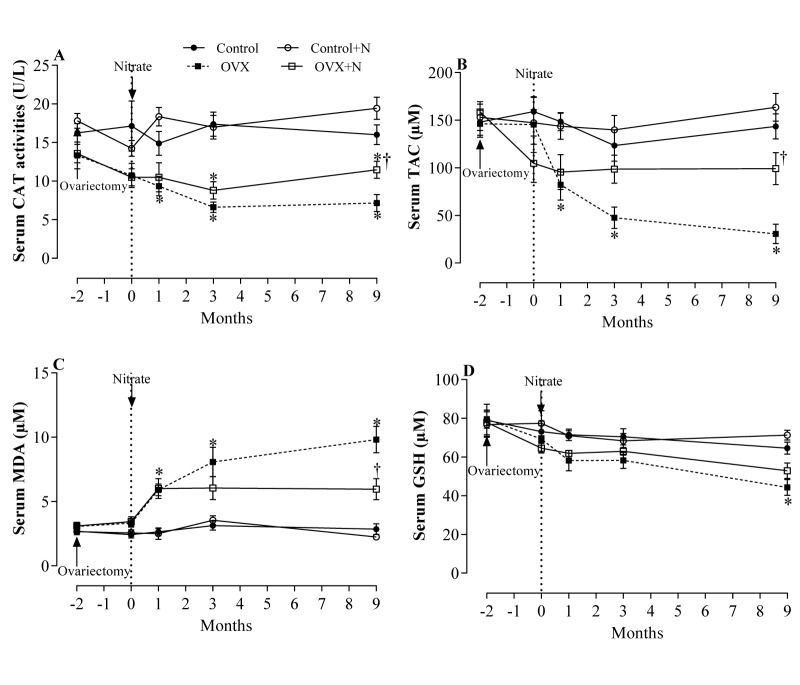
Effects of inorganic nitrate on catalase activity (CAT, A), total antioxidant capacity (TAC, B), malondialdehyde (MDA, C), and reduced glutathione (GSH, D) levels in serum of ovariectomized (OVX) and control rats. ^*, †^ P < 0.05 compared to control and OVX rats, respectively. Results are mean ± SEM (n=6/group).

Compared to the controls, OVX rats had significantly lower TAC levels and higher MDA levels in serum at months 1, 3, and 9. In addition, OVX rats had lower CAT activity in serum at months 3 (P = 0.003) and 9 (P = 0.002) and lower serum levels of GSH at month 9 (P = 0.019). Nitrate administration for 9 months increased serum CAT activity by 60 % (P = 0.027) (Figure 4A(Fig. 4)), TAC levels by 225 % (P = 0.046) (Figure 4B(Fig. 4)), and decreased MDA levels by 39 % (P = 0.098) (Figure 4C(Fig. 4)) only at month 9, but did not affect serum levels of GSH (Figure 4D(Fig. 4)). As shown in Figure 4A-D(Fig. 4), nitrate administration had no significant effect on measured serum oxidative stress indices in control rats.

### Effect of inorganic nitrate on oxidative stress indices in the bone tissue

**Figure 5:**
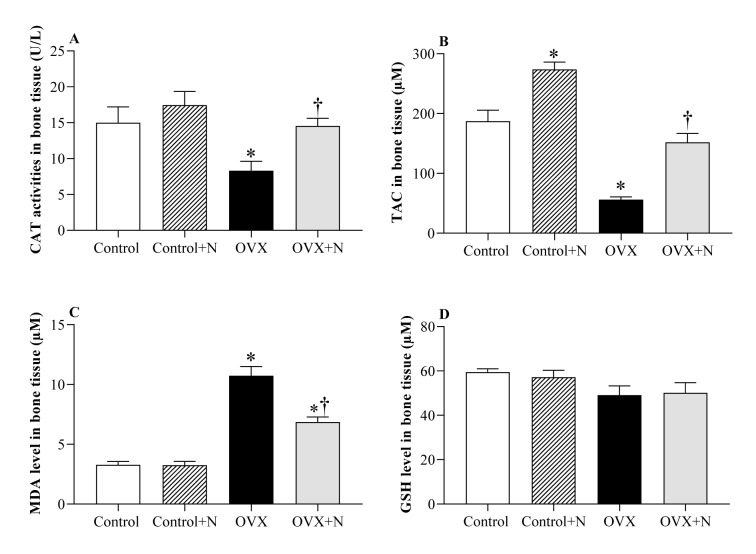
Effects of inorganic nitrate on catalase activity (CAT, A), total antioxidant capacity (TAC, B), malondialdehyde (MDA, C), and reduced glutathione (GSH, D) levels in the bone tissue of ovariectomized (OVX) and control rats. ^*,† ^P < 0.05 compared to control and OVX groups, respectively. Results are mean ± SEM (n=6/group).

Compared to the controls, OVX rats had 45 % (P = 0.064) lower CAT activity, 70 % (P<0.001) lower TAC levels, and 327 % (P<0.001) higher MDA levels in the bone tissue at month 9. Nitrate administration for 9 months increased CAT activity by 75 % (P= 0.096) (Figure 5A(Fig. 5)), TAC levels by 170 % (P < 0.001) (Figure 5B(Fig. 5)), and decreased MDA levels by 36 % (P < 0.001) (Figure 5C(Fig. 5)), while it did not affect GSH levels in bone tissue (Figure 5D(Fig. 5)). In control rats, nitrate administration increased TAC levels by 46 % (P = 0.001) but had no significant effects on other measured parameters.

## Discussion

This study showed that chronic low-dose inorganic nitrate has protective effects against OVX-induced osteoporosis in the proximal tibia of female rats. The anti-osteoporotic effect of inorganic nitrate is associated with blunting OVX-induced NO deficiency and OVX-induced oxidative stress in the circulation and the bone tissue. 

In this study, OVX rats had lower BV/TV (19 %), Tb.N. (33 %), and Tb.Th. (6 %), and higher Tb.Sp. (93 %) from two-month after ovariectomy (8-months-old rats) until the end of the study (17-months-old rats), indicating the occurrence of osteoporosis in the proximal tibia. Similar results have been reported 14 (Wronski et al., 1988[79]; Yousefzadeh et al., 2020[83]), 21 (Conley et al., 2017[15]), 48, 96, and 144 (Liu et al., 2015[37]) days after OVX in the proximal tibia of rats. Indeed 60, 90, and 540 days after ovariectomy, ~50 %, 80 %, and 99 % of the proximal tibia is lost in OVX rats (Wronski et al., 1989[80]; Laib et al., 2001[35]). 

In this study, oxidative stress was higher and eNOS-derived NO production was lower in OVX rats at month 9; eNOS-derived NO deficiency was indicated by lower serum and bone NOx and lower bone eNOS mRNA. In line with our results, decreased serum NOx concentrations, and eNOS mRNA expression (Lirani-Galvão et al., 2009[36]) have been reported 14 (Chou et al., 2010[13]), 28 (van Bezooijen et al., 1998[71]; Stancíková et al., 2004[69]), 56 (Hernández et al., 2000[26]; Ma et al., 2013[40]), and 84 (Hao et al., 2005[25]; Peng et al., 2005[54]; Lirani-Galvão et al., 2009[36]; Guo et al., 2013[23]) days after ovariectomy in rats. In addition, decreased CAT activity and TAC levels and increased MDA levels in serum (Liu et al., 2021[38]) and bone tissue (femur and tibia) have been reported 60 (Aydin et al., 2011[6]), 84 (Aydin et al., 2011[6]; Behr et al., 2012[9]), 91 (Liu et al., 2021[38]), 100 (Arslan et al., 2011[5]), and 140 (Galal et al., 2018[19]) days after ovariectomy in rats; finding that was extended to 330 days after ovariectomy for the first time by our study. It has been reported that estrogen deficiency decreases NOx (Rosselli et al., 1995[61]; Bednarek-Tupikowska et al., 2008[8]) and TAC, while it increases MDA (Kumru et al., 2005[33]; Bourgonje and Abdulle, 2020[12]) in serum that are associated with a higher risk of osteoporotic fractures in postmenopausal women (Sendur et al., 2009[66]) and ovariectomized rats (Muthusami et al., 2005[47]). These data suggest that estrogen deficiency, accompanied by NO deficiency and increased oxidative stress in the bone tissue, contributes to the development of menopause-related osteoporosis. 

In the current study, chronic nitrate administration to the OVX rats increased BV/TV, Tb.N., and Tb.Th. by 42 %, 61 %, and 12 %, respectively, and decreased Tb.Sp. by 15 % at month 9, indicating anti-osteoporotic effects of nitrate in OVX rats. To the best of our knowledge, one study assessing the effects of inorganic nitrate against osteoporosis in OVX rats (Conley et al., 2017[15]) indicates that 3-week administration of inorganic nitrate does not affect tibia microarchitecture in OVX rats. However, anti-osteoporotic effects of nitroglycerine, an organic nitrate, have been documented in the femur following 6 weeks of treatment (Wimalawansa et al., 1996[76]) and in the distal femur and proximal tibia after 4 weeks of exposure (Hukkanen et al., 2003[27]) in OVX rats. In addition, a decreased fracture risk among users of organic nitrates has been reported in case-control studies (Rejnmark et al., 2006[60]; Pouwels et al., 2010[57]) and randomized clinical trials (Wimalawansa, 2000[74]; Nabhan and Rabie, 2008[48]). However, this issue is inconsistent, and some trials reported positive results have been retracted (Bolland et al., 2020[11]). In this regard, according to randomized controlled trials, nitroglycerin does not affect the fracture risk in postmenopausal women after 12 (Bolland et al., 2020[11]) or 36 months (Wimalawansa et al., 2009[77]). Organic nitrates are poorly tolerated and increase the cardiac mortality risk (Nakamura et al., 1999[49]; Bolland et al., 2020[11]); in addition, their anti-osteoporotic effects are lost with increased frequency (Wimalawansa et al., 2000[73]) and duration (Pouwels et al., 2010[57]) of treatment. Inorganic nitrite and nitrate have NO-like effects in the bone (Jeddi et al., 2022[29]) and have been suggested to be appropriate alternatives to organic nitrates (Münzel and Daiber, 2018[46]). Inorganic nitrite and nitrate have simple ionic structures, have more long-lasting effects, are produced endogenously, are present in the diet, and are not limited by the development of tolerance following chronic use (Omar et al., 2012[51]). In support of this suggestion, meta-analyses of observational studies strongly suggest that Mediterranean diet, a rich source of inorganic nitrate, decreases the risk of osteoporotic fractures by 21 % in the general population (Kunutsor et al., 2018[34]; Malmir et al., 2018[41]). 

Our results showed that high trabecular bone quality after nitrate administration in OVX rats is associated with blunting OVX-induced NO deficiency (indicated by increasing serum and bone NOx levels and bone eNOS expression) and blunting OVX-induced oxidative stress in the circulation and the bone tissue. Anti-oxidative effects of inorganic nitrate have been reported in the renal (Gao et al., 2015[20]), liver (Peleli et al., 2015[53]), vascular (Montenegro et al., 2011[45]; Amaral et al., 2019[3]), heart (Donnarumma et al., 2016[16]), and aorta (Sindler et al., 2011[68]) tissues by decreasing the expression and activity of the pro-oxidant enzymes and increasing antioxidant enzymes. Nitrate converts to nitrite and then to NO; NO at low levels (eNOS-derived NO) decreases osteoclast-mediated bone resorption (Percival et al., 1999[55]; Wongdee and Charoenphandhu, 2011[78]) and increases osteogenesis (Yang et al., 2018[82]). Moreover, the anti-osteoporotic effects of estrogen are partly mediated by NO, which is derived from the nitrate-nitrite-NO pathway in the bone (O'Shaughnessy et al., 2000[52]; Rajfer et al., 2019[59]). Therefore, overcoming NO deficiency and the estrogen-like role of nitrate in OVX rats are potential mechanisms by which nitrate can exert its anti-osteoporotic effects in bone tissue.

As for the study strengths, we used 6-month-old rats to induce the osteoporosis model; rats at this age have the best osteoporotic response, lower skeletal growth, and stable serum bone turnover markers (Yousefzadeh et al., 2020[83]). OVX-induced osteoporosis in rats is a clinically relevant model of human postmenopausal bone loss (Kalu, 1991[30]; Yousefzadeh et al., 2020[83]), and according to Food and Drug Administration guidelines, the efficacy of any new treatment for osteoporosis in humans should be verified in OVX rats (Colman, 2003[14]). In addition, OVX-induced bone loss in the proximal tibia, as a region of interest used in the current study, is observed earlier and is more severe than in other bone sites (Francisco et al., 2011[18]; Yousefzadeh et al., 2020[83]) that are clinically similar to humans (Liu et al., 2015[37]). This study has some limitations; we did not assess bone histology to explore the potential protective effects of nitrate against OVX-induced structural damage. In addition, we did not measure genes involved in the osteoblast and osteoclast activity that could determine the possible mechanism involved in the favorable effects of inorganic nitrate against osteoporosis in OVX rats.

In conclusion, chronic low-dose inorganic nitrate administration exerts protective effects against osteoporosis in OVX rats; this effect is associated with increasing eNOS-derived NO and decreasing oxidative stress in the bone tissue. These results indicate that inorganic nitrate therapy may reduce the risk of osteoporotic fractures in NO deficiency conditions such as menopause. These findings are relevant for postmenopausal women since inorganic nitrite/nitrate can be used as a safe and cost‐effective novel therapeutic modality to provide a nutritionally-based interventional protocol against osteoporosis. A randomized trial is necessary to gauge the efficacy of inorganic nitrite/nitrate for treating OVX-induced osteoporosis.

## Declaration

### Acknowledgments

Authors would like to acknowledge the Tehran University of Medical Sciences Preclinical Core Facility (TPCF), Tehran, Iran, for providing the *in vivo* imaging and image processing services for this research.

### Funding information

This study was supported by a grant (Grant No. 98096) from the Research Institute for Endocrine Sciences, Shahid Beheshti University of Medical Sciences, Tehran, Iran.

### Conflict of interest 

The authors declare that they have no competing interests.

### Authorships

NY, SJ, KK, and AG participated in the conception, design, and conduct of the study. NY and AG conducted the statistical analyses. NY and SJ drafted the first version of the manuscript. All authors read and approved the final manuscript.

## Supplementary Material

Supplementary information

## Figures and Tables

**Table 1:**
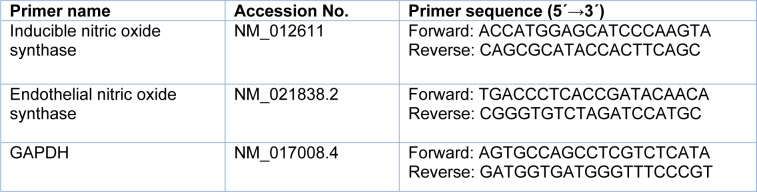
Primer sequences used for real-time PCR

**Table 2:**
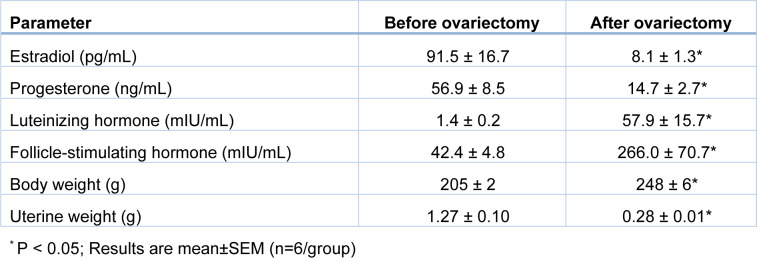
Verification of ovariectomy in rats

**Table 3:**
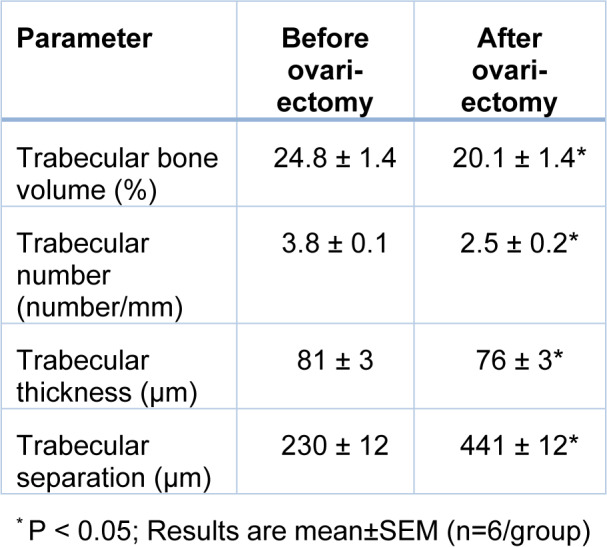
Verification of osteoporosis in rats
