# Supplementary material for: Long-term inorganic nitrate administration protects against ovariectomy-induced osteoporosis in rats
Source: EXCLI J. 2022 Aug 24;21:1151–66. doi: 10.17179/excli2022-5082 (PMC9618708; doi:10.17179/excli2022-5082)
Supplement: Supplementary information [file EXCLI-21-1151-s-001.pdf]

EXCLI Journal 2022;21:1151-1166 – ISSN 1611-2156
Received: May 28, 2022, accepted: August 19, 2022, published: August 24, 2022

Supplementary information to:

Original article:

LONG-TERM INORGANIC NITRATE ADMINISTRATION PROTECTS AGAINST OVARIECTOMY-INDUCED OSTEOPOROSIS IN RATS

Nasibeh Yousefzadeh[1], Sajad Jeddi[1], Khosrow Kashfi[2], Asghar Ghasemi*[1]

[1] Endocrine Physiology Research Center, Research Institute for Endocrine Sciences, Shahid Beheshti University of Medical Sciences, Tehran, Iran
[2] Department of Molecular, Cellular, and Biomedical Sciences, Sophie Davis School of Biomedical Education, City University of New York School of Medicine, NY, USA

* **Corresponding author:** Asghar Ghasemi, Endocrine Physiology Research Center, Research Institute for Endocrine Sciences, Shahid Beheshti University of Medical Sciences, No. 24, Arabi Street, Daneshjoo Blvd, Velenjak, P.O. Box: 19395-4763, Tehran, Iran. E-mail: Ghasemi@endocrine.ac.ir

https://dx.doi.org/10.17179/excli2022-5082

This is an Open Access article distributed under the terms of the Creative Commons Attribution License (http://creativecommons.org/licenses/by/4.0/).

EXCLI Journal 2022;21:1151-1166 – ISSN 1611-2156
Received: May 28, 2022, accepted: August 19, 2022, published: August 24, 2022

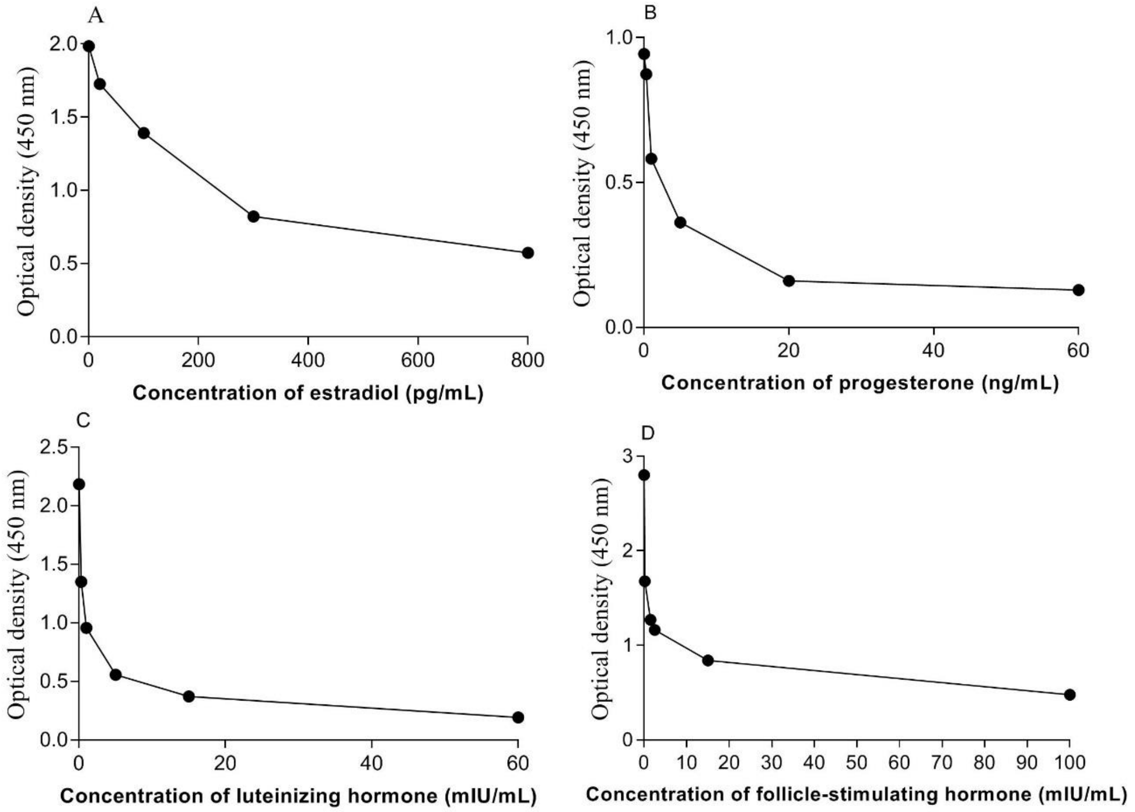

Supplementary Figure 1: Standard curves of estradiol (**A**), progesterone (**B**), luteinizing hormone (**C**), and follicle-stimulating hormone (**D**)

EXCLI Journal 2022;21:1151-1166 – ISSN 1611-2156
Received: May 28, 2022, accepted: August 19, 2022, published: August 24, 2022

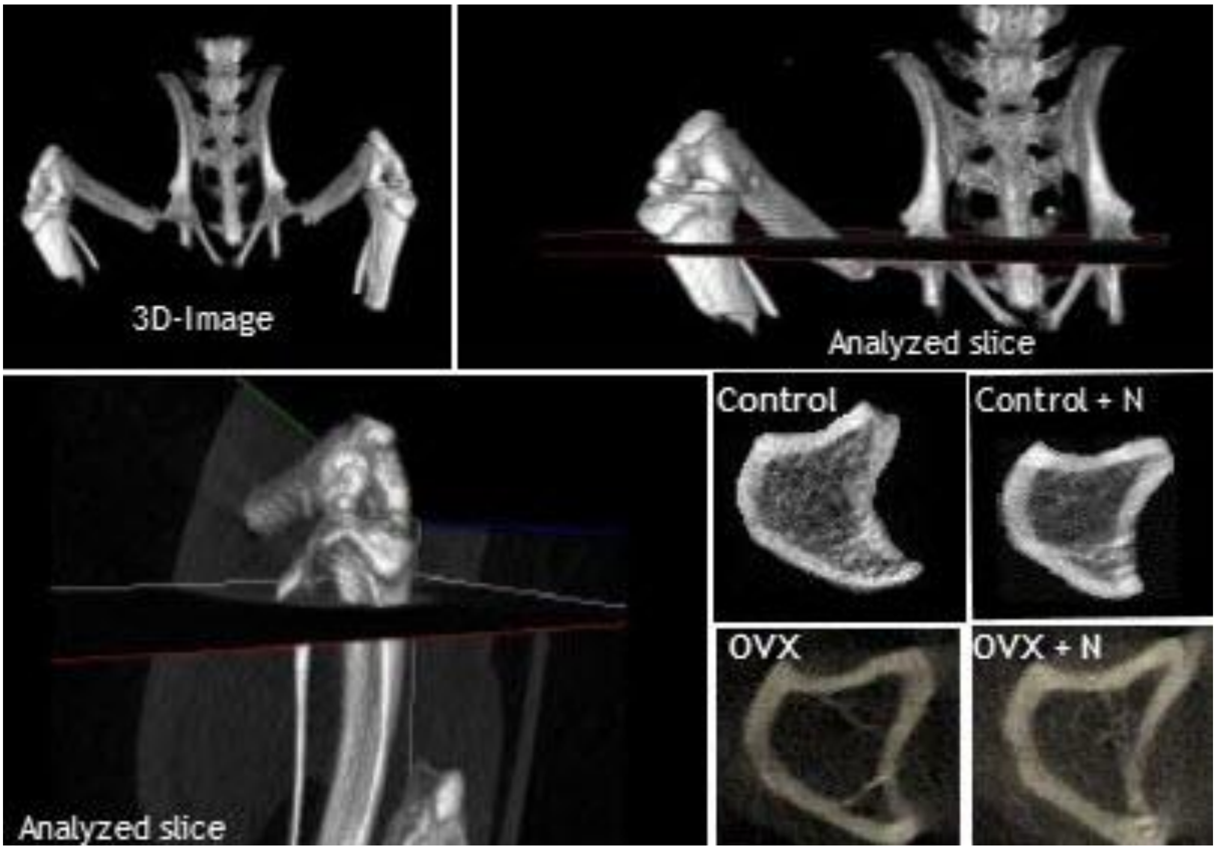

Supplementary Figure 2: Images of micro-computed tomography for proximal tibia in studied groups. Control+N; nitrate treated control rats; OVX+N, nitrate treated ovariectomized rats

Received: May 28, 2022, accepted: August 19, 2022, published: August 24, 2022

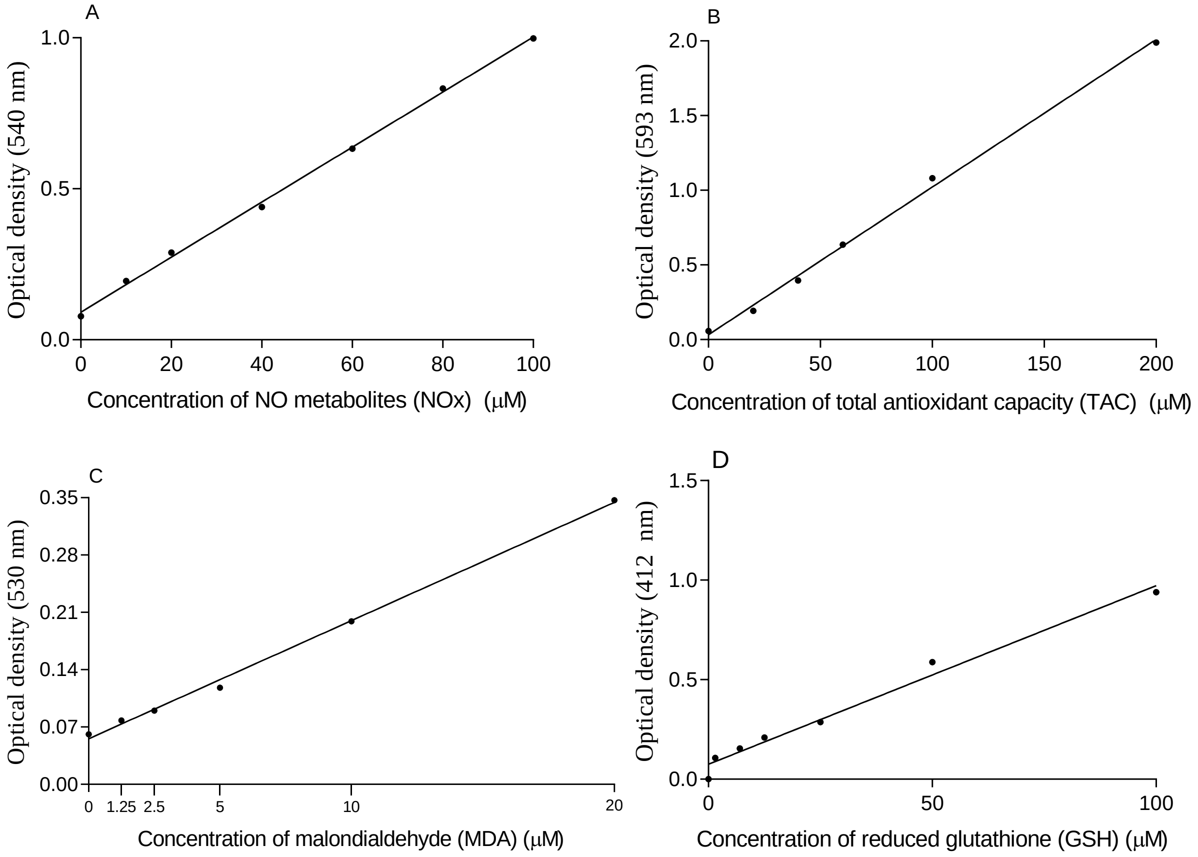

Supplementary Figure 3: Standard curves of nitric oxide metabolite (NOx, **A**), total antioxidant capacity (TAC, **B**), malondialdehyde (MDA, **C**), and reduced glutathione (GSH, **D**)
